# Can the disease course in Parkinson’s disease be slowed?

**DOI:** 10.1186/s12916-015-0534-x

**Published:** 2015-12-10

**Authors:** Amos D. Korczyn, Sharon Hassin-Baer

**Affiliations:** Department of Neurology, Tel Aviv University Medical School, Ramat Aviv, Tel Aviv, Israel; The Movement Disorders Institute, Sagol Neuroscience Center and Department of Neurology, Chaim Sheba Medical Center, Tel-Hashomer, 52621 Ramat-Gan, Israel

**Keywords:** Disease course modification, Disease modifying therapies, Parkinson’s disease, Pathogenesis, Precision medicine, Therapy

## Abstract

**Background:**

The diagnosis of Parkinson’s disease (PD), which is needed for useful symptomatic therapy, is based on clinical criteria. However, it became quite clear in recent years that the same features can occur through different etiopathogenic mechanisms. Even a pathological diagnosis of PD, based on the demonstration of α-synuclein deposits in a typical distribution, can result from different causes and, vice versa, nigral cell loss can occur without α-synuclein deposition.

**Discussion:**

Thus far, attempts to influence the progression of PD have failed. However, since the clinical manifestations of PD can be the result of diverse mechanisms, a single intervention may not be able to slow the course of the disease in all patients. Indeed, targeting the underlying pathogenic processes, which differ among cases, may be more effective. PD may develop as a consequence of mitochondrial damage, which itself may result from a variety of genetic or environmental factors. Correction of the ensuing oxidative stress may theoretically be useful in these PD patients, but will not affect the progression of the disease among other PD patients in whom an identical clinical syndrome derives from defects in other pathways such as the ubiquitin-proteasome system and lysosomal dysfunction, among others.

**Summary:**

Precision medicine can now be used to identify the underlying pathogenic mechanisms in individual patients, paving the way to the development of real disease modification through a pathway-oriented approach, aimed at the underlying biologic processes of disease occurrence and evolution.

## Background

Huge investments in research have dramatically increased our understanding of the biochemical and pathological processes underlying neurodegenerative diseases. Nevertheless, thus far, these advances have failed to be translated into breakthroughs in the treatment of any of these diseases. The impressive progress over the past two decades is the result of improved techniques in neuropathology, genetics and imaging, and the development of animal models. In Parkinson’s disease (PD), specifically, only relatively effective symptomatic treatments are available. Such treatments focus primarily on dopamine-mediated dysfunctions and do not affect the underlying degenerative process, which extends beyond the dopaminergic system. Thus, the impressive accumulation of information regarding the underlying pathology, the molecules involved, and the genetic background causing or contributing to the disease, has not impacted on clinically relevant interventions [1]. The development of neuroprotective therapies that slow, stop, or reverse neurodegeneration in PD has been declared as the single most important unresolved issue in the management of this disorder [2]. Attempts to stop the neurodegenerative process in PD require an understanding of the pathways leading to the selective cell death which characterizes the disease. Over the years, several such processes have been suggested, as detailed below.

It is now widely accepted that sporadic PD, like other neurodegenerative diseases, has a complex pathogenesis involving an interaction of several genetic contributions, each with minor impact, and a number of environmental factors [3]. This understanding could lead to a fatalistic conclusion that it may be impossible to defeat the degenerative process. However, as shown herein, this is not necessarily the case. Rather, it is likely that, at some stage, convergence of several factors occurs leading to a common downstream process (e.g. apoptosis of dopaminergic cells). We maintain that it is only the identification of the pathways leading to this final stage which could allow us to effectively interfere with the pathogenic process.

Recent attempts have been made to target α-synuclein deposition, based on the assumption that α-synuclein plays a causative role in neurodegeneration [4]. It is possible, however, that different processes may lead to α-synuclein accumulation. Moreover, it is unclear whether it is really α-synuclein toxicity which is causing the degeneration [5]. A rational therapy aimed at slowing the progression of the disease (or its prevention) may target α-synuclein, or go beyond it by identifying the processes leading to its misfolding and deposition. Identification of the molecular factors driving the pathogenesis of PD is important in order to define, at the molecular level, not only the disease predispositions but rather the pathogenic processes in each individual patient [6]. However, the fact that several different underlying processes exist, and possibly converge, pose difficulties for the development of a tailored therapeutic regime.

An impressive development in medicine in recent years has been the attempt towards personalized, or individualized, medicine, a term which has now been upgraded to ‘precision medicine’ [7]. Naturally, medicine has always attempted to differentiate subjects based on their background and needs, and to treat them accordingly. In movement disorders, this has resulted in the differentiation between PD, essential tremor, and progressive supranuclear palsy, with important therapeutic implications. Clinical decisions regarding the treatment of PD patients with hypokinetic-rigid syndromes or those with tremor have relied primarily on the clinical phenotype, aiming at symptomatic improvement. Attempts to subdivide PD according to predominant motor symptomatology, age of disease onset, depressive affect, and cognitive performance are based on arbitrary definitions and not on distinct neuropathology [8] and neglect the importance of the underlying pathogenic processes. The therapeutic approach to patients with PD is customized by targeting the symptoms which are most troublesome to the individual patient [9]. Thus, the treatment of tremor-predominant PD patients may differ from that of patients in whom rigidity is the most disabling feature. Furthermore, therapeutic decisions may depend on age, sex, and occupation of the patient, as well as a host of other factors. However, all these considerations only apply to symptomatic therapy. On the other hand, precision medicine attempts to identify the underlying molecular mechanisms responsible for the disease in an individual in order to develop an appropriate therapy. This approach, as applied to PD, will be elaborated below.

The human genome toolbox has expanded beyond the simple identification of mutations or polymorphisms. Transcriptomics, using microarrays and RNA sequencing, can help to identify disease mechanisms, while proteomics, pointing to specific protein profiles, can help in diagnosing different disorders underlying PD. Importantly for the present discussion, metabolomics can be used to identify the underlying cellular metabolic changes occurring in a given disease.

Sporadic PD does not have one single cause, and the clinical features are the result of phenotypic convergence. The separation of the different disorders which manifest similar phenotypes is critically important when attempting to reach and apply precision medicine.

## Discussion

### Genetics of PD

In the past decade, much research has focused on the heredity of PD and the realization of the importance of genetic contributions to the familial forms of PD [10]. The first dominantly inherited form of PD, named as PARK1, is fully (or almost fully) penetrant, and is thought to be related to a toxic effect of α-synuclein or to a loss of functional α-synuclein [5]. In other forms of genetic parkinsonism, like PARK2, α-synuclein may not be involved at all.

However, the majority of PD cases are sporadic. In these, genetic contributions are not clearly detected and, at any rate, are likely to have a marginal role. Genome-wide association studies have provided important information on the genetics of sporadic PD [11], highlighting the implication of several genes and the significant occurrence of population-specific heterogeneity. Most of the identified genes were observed to have a rather small effect and, thus, a large population was needed for their identification [12, 13]. Clearly, several additional genes could contribute to the risk of developing PD, although these could not be detected by studying individual cases.

The genes which have been identified as possibly related to Mendelian forms of PD can be grouped as belonging to at least four or five main classes (Table 1). Each of these classes are defined by a different metabolic pathway, including oxidative stress, lysosomal dysfunction, proteasome-ubiquitin system dysfunction, and inflammation. Independent support of these abnormalities has long been available [14–17].

**Table 1:** Pathogenic processes underlying Parkinson’s disease and presumed related genetic loci, genes, and proteins

	Disease	Gene locus	Gene	Encoded protein
Mitochondrial dysfunction and oxidative stress	PARK7 PARK6	1p36 1p36	*DJ-1* *PINK1*	DJ-1 PTEN-induced kinase 1
Neuroinflammation and immune mechanisms	PARK2 PARK1/4	2p13 6q26 4q22.1	*PARK3* *PARK2* *SNCA*	Parkin α-synuclein
Altered protein handling (including the ubiquitin*-*proteasome system)	PARK5 PARK17	2p13 4q14 16q11.2	*PARK2* *UCHL1* *VPS35*	Parkin Ubiquitin carboxy-terminal hydrolase L1 Vacuolar protein sorting-associated protein 35
Lysosomal dysfunction	Gaucher’s related PARK9	1q22	*GBA* *ATP13A2*	Glucocerebrosidase ATPase 13A2
α-synuclein aggregation	PARK8 PARK1/4	12q12 4q22.1	*LRRK2* *SNCA*	Leucine-rich repeat kinase 2 α-synuclein

The low risk associated with each of the genes involved in the pathogenesis of sporadic PD likely indicates that a convergence of factors, some genetic and others environmental, is needed to trigger the disease. Potentially, dozens of risk alleles, each having a minute effect, are cooperating to affect the liability to disease, and therefore the identification in a given person of one haplotype or another is not likely to be helpful in either estimating the risk of developing sporadic PD or its rate of progression and is thus an unlikely target for intervention. In order to affect the neurodegenerative process, it is necessary to identify the biochemical process to which each gene contributes. Monogenic diseases suggest that separate processes may occur in different PD patients (Table 1).

### Genomics

Information from genomics is theoretically therapeutically useful in cases having primarily a strong genetic background, particularly those with Mendelian inheritance. For example, genetic manipulation could replace a mutated α-synuclein gene by a normal one. Although this is still not applicable in humans, there is no apparent theoretical reason why this could not be performed, or why an abnormal α-synuclein gene could not be silenced [18]. This technique has already been performed in experimental animals. Thus, Kachroo and Schwarzschild [19] reported that disruption of the adenosine *A2A* receptor gene may protect experimental animals from dopaminergic cell loss induced by α-synuclein overexpression. Such an intervention, if and when successfully implemented in humans, could shift the focus from reactive to preventive healthcare. However, while this therapy would be relevant to individuals carrying a duplicated (and perhaps mutated) α-synuclein gene, it would not be relevant to most PD patients. In cases with a genetic background, the situation is more complex. For example, for *LRRK2* mutation carriers, it is not clear what the underlying molecular process responsible for the disease is, and whether in a given disease-associated genotype the mutated gene leads to increased enzymatic kinase activity or reduces it, let alone which molecules phosphorylated by LRRK2 are relevant to the development of PD.

### Epigenomics

The complexity of sporadic PD is underlain by the fact that the disease is caused in different people by a variable contribution of genetic polymorphisms and environmental factors. Environmental factors might exert their effect in several ways, for example, through a direct toxic effect (e.g. agricultural pesticides or herbicides). However, it is increasingly recognized that these environmental factors might operate by modifying genetic pathways. Rather than altering DNA itself, these factors may operate epigenetically, by changing the expression of certain genes. The field of epigenetics attempts to explore the mechanisms by which environmental factors interact with genetic expression. These epigenetic effects may occur at any age, but older people are more vulnerable. For instance, oxidative DNA damage increases in the aged due to the reduced efficiency of repair enzyme systems. This may explain the fact that, even in cases with a genetic background, PD manifests itself typically in middle life or later. Presumably, even in cases with Mendelian dominant or recessive inheritance, a ‘second hit’ is needed, with environmental stressors perhaps exerting an epigenetic effect.

### Biomarkers

Biomarkers can include changes in body chemistry, anatomy, or physiology in genes and how they are regulated, and even subtle differences in a person’s behavior, but may also serve as important clues to the etiology of the disease. For example, certain antibodies in the blood can be biomarkers for different types of infections. In recent years, several attempts have been made to identify biomarkers for PD [20]. Acknowledging the difficulties in diagnosing PD based on clinical criteria, biomarkers could help in several ways, for example, in the prognostication or inclusion into therapeutic studies, but also in detecting early clinical and even preclinical cases and allowing early intervention. The attempt to identify such biomarkers has traditionally been based on the assumption that PD is etiologically homogeneous. As mentioned above, this assumption may be wrong. If what is presently included under the PD umbrella are clinical phenotypes originating in different mechanisms [21], the failure to find such biomarkers is not surprising. If indeed PD is etiologically and pathogenetically heterogeneous, biomarkers, which will be applicable to all cases, if identified, are likely to indicate downstream changes.

### Metabolomics

Several biochemical/metabolic pathways, which may lead to an identical (or very similar) clinical PD phenotype, have been suggested over the years. These include abnormalities of mitochondrial function or oxidative stress in several genetic forms (e.g. *PINK1*) [22], while in others, impaired protein degradation (ubiquitin-proteasome system, as in *PARKIN* mutation carriers) [23] or lysosomal dysfunction (as in *GBA* mutation carriers; Table 1) [24] may occur. Metabolomics research is important because it can establish the relevant biochemical pathway involved in disease pathogenesis in a given patient regardless of the primary cause, which can help to identify interventions which could modulate these pathways. The metabolomic approach is obviously helpful in patients with monogenic disease pointing to the specific effect of the mutation. For example, the dysfunction by which *SNCA* changes cause PD has not been deciphered, although indications of protein mishandling, mitochondrial damage, and oxidative stress, as well as lysosomal dysfunction and inflammation have been suggested [25]. Thus, the identification of *SNCA* mutations by itself still does not lead to identification of the relevant mechanisms, which is required in order to develop a specific therapy against α-synuclein-triggered neurodegeneration. Since it is difficult to find the exact molecular mechanism through which this (and other) mutations operate, it could be useful to find intermediate steps, neither high upstream nor low downstream. This approach could be particularly useful in those patients with a complex disease pathogenesis where a constellation of genetic polymorphisms and environmental factors contribute to disease pathogenesis.

Lysosomal dysfunction has been identified as an important mechanism of the pathogenesis of PD associated with GBA mutations [26, 27] and other genetic forms [28], but it has also been suggested to be involved in sporadic PD [29].

Mitochondrial dysfunction has long been suspected to be involved in PD pathogenesis [30–33]. Nevertheless, if mitochondrial dysfunction only applies to a subgroup of patients, this has important clinical implications. Treatment with Coenzyme Q-10 (a naturally occurring antioxidant that affects mitochondrial depolarization and acts as an electron transporter for mitochondrial complexes I and II) has been studied in PD based on the assumption that this drug may ameliorate neuronal oxidative stress [34, 35]. Additional approaches to this target have recently been suggested [36]. However, clinical studies with pioglitazone have been disappointing [37], possibly since they included a heterogeneous PD population. A more appropriate approach could have been to focus on those individuals with evidence of mitochondrial dysfunction, who are the rational targets for correcting this pathology.

Inflammatory changes have also been proposed to be associated with the pathophysiology of PD [16, 38–40] and, while the importance of this process is not well understood, it is plausible that it also differs among individual patients. Thus, in those subjects in whom inflammation is exerting a significant impact on the disease mechanism, attempting to quench it could be rewarding.

Metabolomic therapy, aimed at the presumed pathogenic mechanism, should have disease-modifying properties, slowing or stopping disease progression, but may look completely inefficacious if the selected endpoints in a study are short-term symptomatic effects. Demonstration of the usefulness of the metabolomic approach was provided recently by Johansen et al. [41], who showed that the metabolomic profile of PD patients carrying the *LRRK2* G2019S haplotype differed from that of cases with sporadic PD. To some extent, *LRRK2* G2019S carriers with PD also differed from clinically unaffected carriers.

### Therapeutic implications

An attempt to identify a single disease-modifying therapy which will benefit all PD patients may be naïve, as different patients may have completely different underlying processes. Therefore, if specific interventions are to be developed, the pathogenic process leading to PD needs to be understood for each individual; this is the concept of precision medicine [7]. Sporadic cases are assumed to result from a combination of polygenic inheritance, interacting with a variety of environmental factors (some possibly protective, e.g. smoking, coffee or tea drinking [42] whilst others damaging, e.g. head trauma), acting in a concerted way. The implications of precision medicine are that, in terms of neuroprotective therapy at least, one should move ahead from phenotype diagnosis to characterization of the underlying pathogenetic process in each individual patient. This could lead to a rational approach to develop a disease-modifying therapy.

### Summary

PD is a complex disorder and the clinical manifestations of sporadic PD are likely to be due to phenotypic convergence, where the disease is caused by multiple contributions of genetic and environmental factors that may differ in each patient. It is therefore not surprising that animal studies have failed to find a disease-modifying therapy for the human disease. Such studies typically employ a single animal strain, with a distinct and unique pathogenesis, and therefore they cannot be expected to be relevant to sporadic PD patients in whom the disease develops through different pathogenetic pathways.

The enormous advances made in biology, genomics, transcriptomics, proteomics, and metabolomics allow a fresh exploration of neurodegenerative diseases and illuminate PD pathogenesis, facilitating a pathway-oriented approach which may provide individual prognostication and intervention to affect disease progression.

The realization that PD is not a disease but rather a syndrome, defined by its clinical phenotype and caused by several distinct mechanisms [18, 36], has several logical consequences. Firstly, attempts to identify biomarkers for the ‘disease’ will either fail or identify processes in the final stages of the disease, e.g. apoptosis, which are of limited clinical relevance. Similarly, it is possible that toxic substances only affect susceptible people. For example, a mitochondrial toxin is more likely to damage individuals in whom mitochondrial function is (genetically) compromised. More importantly, development of disease-modifying therapies depends on identifying the underlying pathogenesis; it is unlikely that any such therapy will be discovered if different pathogenetic mechanisms operate in different individuals, as long as the study would include a heterogeneous combination of cases. The development of such a disease-modifying therapy would require the identification of biomarkers for the pathogenesis, e.g. oxidative stress, the selection of patients with this form of pathogenesis, and their treatment with a relevant drug, e.g. an anti-oxidative drug. Similar suggestions have been recently brought forward [39]. In the era of personalized medicine [7], an attempt must be made to identify early changes. These could include genetic polymorphisms or epigenetic factors, each of which can lead to specific therapies, e.g. pharmacogenomic interventions.

Finally, it is possible that the different pathogenic mechanisms seen in monogenic PD cases are closely interacting, for example, protein mishandling affects mitochondrial dysfunction and vice versa. If that is the case, there could still be a single predominant pathogenic mechanism regarded as the ‘primary’ step in a given individual. Otherwise, disease modification will require concerted action in several fronts, acting on several pathogenic processes simultaneously, such as by multifunctional therapies or drug cocktails. If this is the case, the prospects of developing disease-modifying therapies are considerably diminished.
